# Design and Analysis of a Bionic Gliding Robotic Dolphin

**DOI:** 10.3390/biomimetics8020151

**Published:** 2023-04-10

**Authors:** Yang Zhang, Zhengxing Wu, Jian Wang, Min Tan

**Affiliations:** 1 School of Artificial Intelligence, University of Chinese Academy of Sciences, Beijing 100049, China; zhangyang2020@ia.ac.cn (Y.Z.); jianwang@ia.ac.cn (J.W.); min.tan@ia.ac.cn (M.T.); 2 The Laboratory of Cognitive and Decision Intelligence for Complex System, Institute of Automation, Chinese Academy of Sciences, Beijing 100190, China

**Keywords:** gliding robotic dolphin, bionic robot, underwater gliding motion, dynamic model

## Abstract

In this paper, we focus on the design and analysis of a bionic gliding robotic dolphin. Inspired by natural dolphins, a novel bionic gliding robotic dolphin is developed. Different from the existing ones, the gliding robotic dolphin developed in this work is specially introduced with a yaw joint to connect its three oscillating joints to improve maneuverability in both dolphin-like swimming and gliding motion. Consequently, the gliding robotic dolphin can realize several flexible motion patterns under the coordination of its flippers, yaw joint, oscillating joints, and buoyancy-driven modular. Thereafter, relying on the Newton–Euler method, a hybrid-driven dynamic model is constructed to further analyze the propulsive performance in both dolphin-like swimming and gliding motions. Finally, various simulations and experiments, including forward swimming, gliding, and turning in both dolphin-like swimming and gliding modes, are carried out to validate the effectiveness of the developed gliding robotic dolphin.

## 1. Introduction

The oceans are the cradle of life and cover nearly three-quarters of the Earth’s surface. They provide humans with many resources and services, such as oxygen, climate regulation, carbon sequestration, food and medicine, and are important for the survival and development of human society. In recent years, marine exploitation activities, represented by seabed surveys, sampling, and seabed operations, have attracted research interest in myriad aspects. Benefiting from the capability of independently completing pre-programmed missions, autonomous underwater vehicles (AUVs) are playing an increasingly significant role in ocean exploitation [1]. However, conventional AUVs suffer from various limitations, e.g., strong noise, poor maneuverability, and slow speed. As a result, the research interest in bio-inspired underwater robots is gradually growing [2,3,4]. Bionic underwater robots perform better in terms of mobility and efficiency compared to conventional robots [5]. For example, the robotic dolphins, which have the capacity to perform a variety of complex motions, such as the leaping action and 360${}^{\circ }$ frontflip–backflip motion, stand out from conventional AUVs [6,7]. However, the excessive energy consumption due to the maneuverable motion considerably hinders its practical application. Inspired by conventional underwater gliders, a gliding mechanism was introduced into the robotic dolphin, thus the gliding robotic dolphin is developed [8]. The gliding motion consumes very little energy [9,10] and helps to extend the endurance of the robotic dolphin. After that, many gliding robotic dolphins have been developed, e.g., a 1.5-m-long robotic dolphin [11]. Superior to the conventional underwater gliders, the gliding robotic dolphins are equipped with controllable surfaces to cope with heading and pitch control issues [11].

In nature, dolphins’ swimming kinematics are characterized as thunniform swimmers. The swimming motions consist of oscillations where bending is restricted to particular points along the body [12,13]. Although cetaceans have a morphology that helps to enhance stability [12], many dolphins can perform outstanding turning maneuverability, e.g., bottlenose dolphins can realize small radius turns with a mean radius of 0.2 (Body Length, BL) and a maximum turning rate of 561.6${}^{\circ }$/s [14] and a killer whale can realize a turning motion with a turning radius of 0.11 BL and a turning rate of 232.5${}^{\circ }$/s [15]. For underwater robots, maneuverability is an essential performance metric. In order to obtain sufficient lift force, the flippers and body segments of conventional gliding robotic dolphins are horizontally set, which limits the turning maneuverability. Because their turning moments can only be generated by deflecting one of the flippers. Due to the short arm of the force, this method leads to insufficient turning moments, that is, their maneuverability is quite limited. Many researchers have devoted a great deal of effort to solve the problem. For instance, Liu et al. analyzed the turning patterns of a dolphin robot based on the bilateral flippers and a small turning radius was realized [16]. Wang et al. improved the turning maneuverability of their robotic dolphin by adopting a rudder on its belly [17]. For the previous robotic dolphins, adopting the body and/or caudal fin (BCF) mode performs poorly in the aspect of turning maneuverability. So, most of the research interest focuses on the turning patterns based on median and/or paired fin (MPF) mode. Natural dolphins can achieve flexible turning with multiple degrees of freedom at the waist and tail joint. Consequently, improving the maneuverability of the bionic gliding robotic dolphin is a very important issue.

In this paper, we develop a novel bionic gliding robotic dolphin system by fusing gliding and dolphin-like oscillation motions. Compared with the existing gliding robotic dolphins [11,17], a special yaw joint is introduced into the mechanism design to improve the maneuverability of the robot in both dolphin-like swimming and gliding motions. For the newly updated bionic gliding robotic dolphin, a corresponding dynamic model involving both gliding motion and dolphin-like oscillation motion is constructed based on Newton–Euler dynamic method. Simulations and underwater experiments have verified that the bionic robotic gliding dolphin has good locomotion capabilities. Simultaneously, the effectiveness and accuracy of the proposed model are validated by comparing and analyzing the simulation and experimental data of the bionic gliding robotic dolphin.

The rest of this paper is organized as follows. In Section 2, a brief description of the mechanical structure design of the bionic gliding robotic dolphin is provided. Section 3 describes the dynamic model and simulation results of the robotic dolphin. The experiment results are discussed in Section 4. Finally, the conclusions and future work are summarized in Section 5.

## 2. Mechanical Design

**Figure 1:**
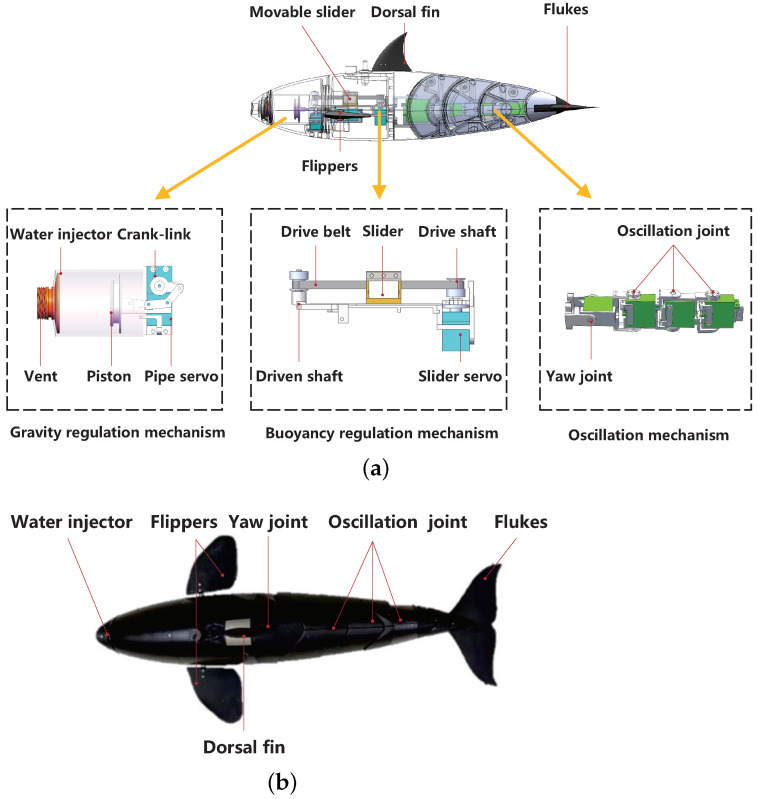
The developed bionic gliding robotic dolphin. (**a**) Mechanical structure; and (**b**) prototype.

The overall design scheme of the bionic gliding robotic dolphin was illustrated in Figure 1. Its streamlined body shape mimics the killer whale, the largest species of Delphinidae, and scales down to 600 mm in length, 105 mm in width and 70 mm in height. The internal mechanism arrangement is highly compact to achieve a tiny volume size. In the end, a 2.8-kg bionic gliding robotic dolphin is designed, which is perfectly formed with both a dolphin-like module and a glide module.

The dolphin-like module is composed of a dorsoventral oscillation mechanism, a pair of flippers, and a yaw joint. The dorsoventral oscillation mechanism employs a three-joint motion structure driven by three servo motors. Similarly, the flipper mechanism and yaw joint are also powered by servo motors. In order to realize the dolphin-like swimming, a motion controller based on the central pattern generator (CPG) is adopted to generate the rhythmic signals [18,19,20]. Under the governance of the generated rhythmic signals, the robotic dolphin can realize both BCF locomotion and MPF locomotion for forward swimming, turning, floating and diving. For example, based on the controllable flippers, the bionic robotic dolphin can obtain pitch and yaw moments by synchronizing and differentially rotating the two flippers, respectively, to realize various motions. Between the oscillating joint and the head of the bionic gliding robotic dolphin, a yaw joint is mounted. The yaw joint is driven by a servo, which can acquire offset angles ranging from −40${}^{\circ }$ to 40${}^{\circ }$. The turning ability of the bionic gliding robotic dolphin can be significantly enhanced by the aid of the yaw joint. Therefore, the inserted yawing joint mechanism makes the developed bionic gliding robotic dolphin have a turning ability that is better than the existing ones [11,17].

As shown in Figure 1a, there are an injector-piston buoyancy regulation mechanism and a slider-mass gravity regulation mechanism for the gliding motion. In particular, the buoyancy regulation mechanism is used to regulate the net buoyancy of the bionic gliding robotic dolphin. At the same time, the gravity regulation mechanism modulates the position of the center of gravity to adjust the body attitude. These two components make up the gliding module, which guarantees that the bionic gliding robotic dolphin stays at a proper glide angle to achieve gliding motions. The buoyancy regulation mechanism consists of a servo motor, a set of toggle links with a piston and a water injector. The injector has a diameter of 49 mm and a height of 55 mm for water storage, about 104 mL in volume. It means the water filled in the injector weighs about 104 g, which is approximately 3.7% of the total displacement of the bionic gliding robotic dolphin. A servo motor is employed as a driver to change the position of the piston. The servo-generated excitations are transferred via toggle links. The slider mass is driven by a belt drive mechanism. The slider is made of a tungsten–copper alloy, weighs about 232 g and has an effective sliding range of 40 mm. It can provide a maximum pitching moment of $9.28\times 10^{-3}$ N·m. As a result, the volume of water in the injector can be precisely changed by the servo motors to obtain high-accuracy net buoyancy control. In the same way, the servo motor precisely controls the position of the slider mass.

## 3. Dynamic Modeling

An accurate dynamics model is an essential tool for future studies on motion control of bionic gliding robotic dolphins. In the course of the simulation, various issues will be recovered in time via an accurate dynamic model [21,22,23]. In this way, the efficiency of research will be substantially improved. Hence, the developed dynamic model compensates for the deficiencies of the current existing studies of the gliding robotic dolphin and lays the foundation for the design and validation of subsequent multimodal motion control methods.

**Figure 2:**
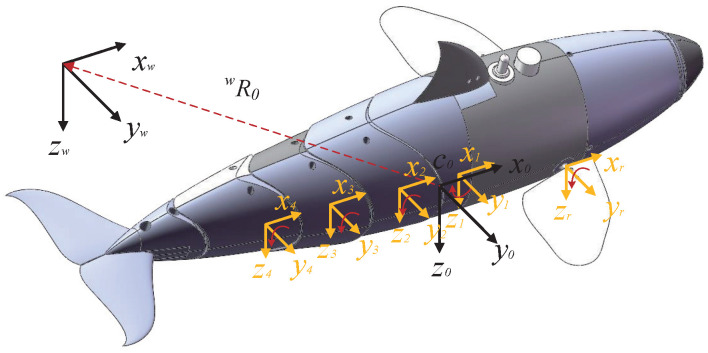
The coordinate systems of the gliding robotic dolphin.

First of all, the definitions of the notations are present as follows. As is shown in Figure 2, the coordinate system consists of an inertial frame $C_{w}=\left(o_{w},x_{w},y_{w},z_{w}\right)$, a body-fixed frame $C_{0}=\left(o_{0},x_{0},y_{0},z_{0}\right)$, and also the frame $C_{i}=\left(o_{i},x_{i},y_{i},z_{i}\right)$ fixed on body parts (i=1,2,3,4), and two flippers (i=l,r). Moreover, the vector ${}^{w}V$ represents the generalized velocity of the robotic dolphin with respect to the body-fixed frame $C_{0}$. Then, we transform ${}^{w}V$ into a velocity vector with regard to the inertial coordinate system $C_{w}$. The process is presented as follows:

(1) $${}^{w}V=\left(\begin{array}{c} U_{w}\\ \Omega_{w} \end{array}\right)=\left(\begin{array}{c} {}^{w}R_{0}U_{0}\\ {}^{w}R_{0}\Omega_{0} \end{array}\right)$$

where ${}^{w}R_{0}$ denotes the transformation matrix from frame $C_{0}$ to frame $C_{w}$, $U_{w}$ and $\Omega_{w}$ denote the three-dimensional velocity and angular velocity vectors in $C_{w}$, respectively, $U_{0}$ and $\Omega_{0}$ denote the three-dimensional velocity and angular velocity vectors in $C_{0}$, respectively. Note that the center of buoyancy of the robotic dolphin is located at the origin of $C_{0}$ and the center of gravity is located 4 mm below the center of buoyancy.

The kinematics of the gliding robotic dolphin are shown below, taking into account the yaw and flipper surfaces:

(2) $$\begin{array}{c} \begin{array}{c} \begin{array}{c} \;V_{i}={}^{i}T_{i-1}V_{i-1}+{\dot{\theta }}_{i}\left[\begin{array}{cc} 0_{1\times 5} & 1 \end{array}\right]\\ \;={}^{i}T_{i-1}V_{i-1}+{\dot{\theta }}_{i}Z\\ {}^{i}T_{i-1}=\left(\begin{array}{cc} {}^{i}R_{i-1} & -{}^{i}R_{i-1}{}^{i-1}{\hat{P}}_{i}\\ 0_{3\times 3} & {}^{i}R_{i-1} \end{array}\right) \end{array} \end{array} \end{array}$$

(3) $$\begin{array}{c} V_{r/l}={}^{r/l}T_{0}V_{0}+{\dot{\theta }}_{r/l}Z\\ {}^{r/l}T_{0}=\left(\begin{array}{cc} {}^{r/l}R_{0} & -{}^{r}R_{0}{}^{0}{\hat{P}}_{r/l}\\ 0_{3\times 3} & {}^{r/l}R_{0} \end{array}\right) \end{array}$$

where $V_{i}=\left[U_{i};\Omega_{i}\right]$ denotes the velocity vector in $C_{i}$, ${}^{i}T_{i-1}$ is the homogeneous transformation matrix of $C_{i-1}$ with respect to $C_{i}$, $\theta_{i}$ denotes the angular velocity of joint *i*, ${}^{i}R_{i-1}$ is the rotation matrix of $C_{i-1}$ with respect to $C_{i}$, ${}^{i-1}{\hat{P}}_{i}$ is a skew symmetric matrix of the position vector of $C_{i-1}$ with respect to $C_{i}$, $V_{r/l}=\left[U_{r/l};\Omega_{r/l}\right]$ denotes the velocity vector of the right (or left) flipper in $C_{r/l}$, ${}^{r/l}T_{0}$ is the homogeneous transformation matrix of $C_{0}$ with respect to $C_{r/l}$, $\theta_{r/l}$ denotes the angular velocity of the right (or left) flipper, ${}^{r/l}R_{0}$ is the rotation matrix of $C_{0}$ with respect to $C_{r/l}$, ${}^{0}{\hat{P}}_{r/l}$ is skew symmetric matrix of the position vector of $C_{r/l}$ with respect to $C_{0}$.

In the previous work [17], we have provided a detailed dynamic modeling of a gliding robotic dolphin with only two oscillating joints. The gliding robotic dolphin developed in this work introduces a yaw joint to increase the turning capability. Meanwhile, three oscillating joints are employed for propulsion. Similarly, we can easily obtain the dynamic model of the gliding robotic dolphin involving both dolphin-like and gliding modes, based on the Newton–Euler dynamic method. Here, we directly give out the dynamic models of the gliding robotic dolphin, and for details, readers can refer to our previous work [17].

(4) $$M{\dot{V}}_{0}=F_{c}+F_{h}+G_{0}+F_{m}+F_{s}-F_{e}$$

$$\begin{array}{cc} & M=\sum_{i=1,2,3,4,l,r}^{}{}^{0}T_{i}M_{i}{}^{i}T_{0}\\ & F_{c}=-\sum_{i=1,2,3,l,r}{}^{0}T_{i}F_{ci}\\ & F_{h}=\sum_{i=1,2,3,l,r}{}^{0}T_{i}F_{hi}\\ & F_{m}=m_{m}\left(\begin{array}{c} 2{\hat{\dot{r}}}_{m}\Omega_{0}-{\ddot{r}}_{m}\\ {\hat{r}}_{m}\left(2{\hat{\dot{r}}}_{m}\Omega_{0}-{\ddot{r}}_{m}\right) \end{array}\right)\\ & F_{s}=m_{s}\left(\begin{array}{c} 2{\hat{\dot{r}}}_{s}\Omega_{0}-{\ddot{r}}_{s}\\ {\hat{r}}_{s}\left(2{\hat{\dot{r}}}_{s}\Omega_{0}-{\ddot{r}}_{s}\right) \end{array}\right) \end{array}$$

where $F_{s}$ and $F_{m}$ stand for the force and torque generated by the water injector and the movable slider, respectively; $F_{hi}={\left(f_{hi},\tau_{hi}\right)}^{T}$ indicates hydrodynamic force and torque of part *i*; $F_{ci}={\left(f_{ci},\tau_{ci}\right)}^{T}$ is defined as the Coriolis force and centripetal force of each part; $F_{i-1,i}={\left(f_{i-1,i},\tau_{i-1,i}\right)}^{T}$ is the external force of part i−1 acting part *i*; thus, there exists the same interpretation of $F_{i,i-1}={\left(f_{i,i-1},\tau_{i,i-1}\right)}^{T}$. Additionally, $G_{0}={\left(G_{n},\tau_{n}\right)}^{T}$ is the net buoyancy force and torque, and the total inertia matrix is defined as $M_{i}$, with $P_{i}$ representing the position vector of part *i*.

**Figure 3:**
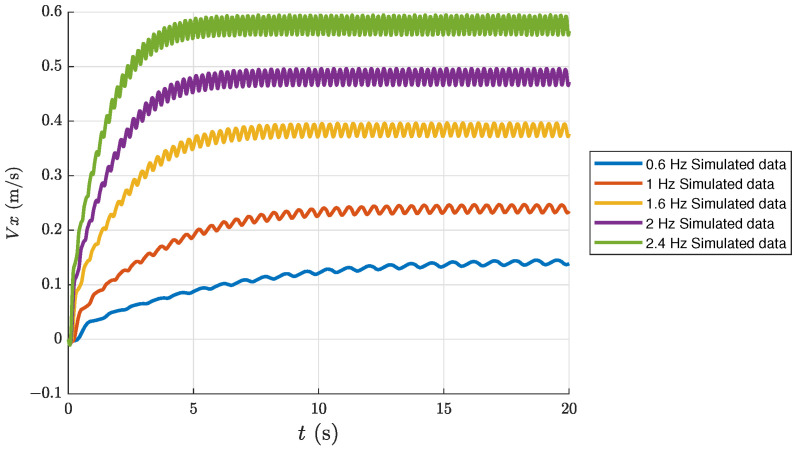
Simulated velocities under different frequencies of the dolphin-like forward swimming.

**Figure 4:**
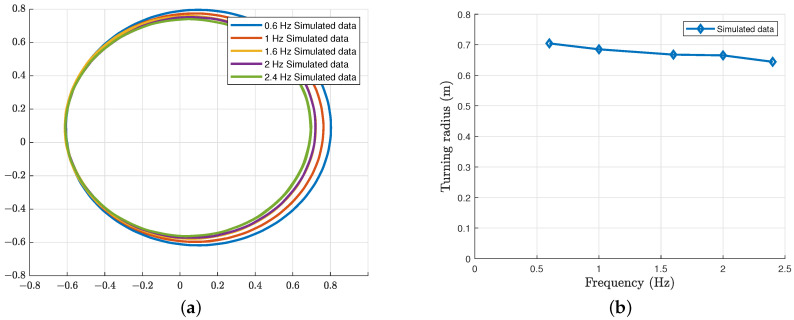
Simulation results of the dolphin-like turning with the yaw joint. (**a**) Swimming trajectory under different frequencies; and (**b**) Turning radius under different frequencies.

In order to validate the dynamic model, we carried out some simulations, involving the dolphin-like forward swimming and dolphin-like turning, as shown in Figure 3 and Figure 4. In particular, Figure 3 shows some results of the dolphin-like forward swimming under different frequencies. We can see that the forward velocity gradually increases and stabilizes at a fixed value after a period of time. As the oscillation frequency increases, the steady-state velocity also increases, respectively. Figure 4 shows the simulation results of the condition of turning with the yaw joint. With the same yaw angle of the yaw joint, the trajectory radius decreases correspondingly as the oscillation frequency rises.

## 4. Experiments Results and Analyses

The dynamic model developed above is built in an ideal state and the physical parameters used are also theoretical values calculated by Solidworks. However, there are many uncertainties and disturbances in practical applications, so the accuracy of the model needs to be experimentally verified. In the following, we carried out several experiments to validate the performance of the developed bionic gliding robotic dolphin as well as the effectiveness of the built dynamic model.

### 4.1. Experimental Setup

**Figure 5:**
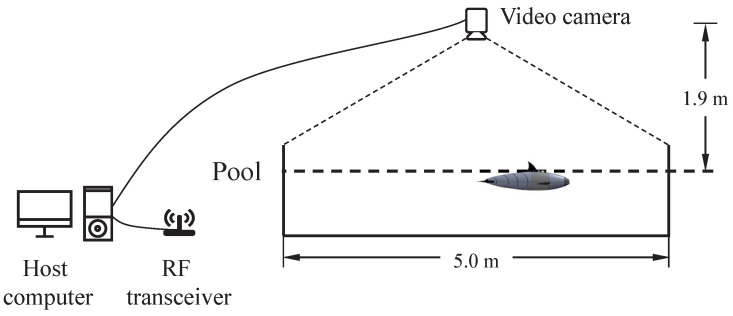
Schematic of the motion measurement system.

The basic experiments of the bionic gliding robotic dolphin were completed in an indoor pool. As is shown in Figure 5, the locomotion of the robot can be recorded and identified with the help of a global camera placed above the pool and self-mounted sensors, which were used to analyze and study the basic swimming performance of the bionic gliding robotic dolphin. The movement of the bionic gliding robotic dolphin is governed by an embedded controller (master chip: STM32F407) located in the head, which runs the core control algorithms to generate control signals and also receives commands or sends data via a radio frequency (RF) transceiver. After receiving the swimming orders from the host computer, the robotic dolphin can swim freely in the pool. In order to assist in distinguishing the target from the background, the bionic gliding robotic dolphin head is affixed with red and yellow labels. The indoor pool size is 5 m × 4 m × 1.3 m (length × width × depth). The global camera resolution is 1292×964, and the maximum recording frame rate is 30 FPS (frames per second). In order to reduce the influence of water reflection, the light source in the experimental environment should be shielded to avoid the direct light on the water surface. However, it also dims the ambient light, which the camera has to compensate for with a longer exposure. Generally, the camera works at around 22 FPS for adequate exposure. Contrastive analysis was implemented under different swimming modes of the bionic gliding robotic dolphin and the consequences illustrated that the dynamic model is credible.

Initially, by analyzing the simulation results, the parameters with the best performance were selected to carry out the experiment. The experiments include forward swimming in dolphin-like mode with different oscillation frequencies, turning with the aid of the yaw joint, and gliding motions. In particular, the oscillation frequencies chosen for the dolphin-like mode are 0.6 Hz, 1 Hz, and 1.6 Hz. The yaw joint offset angle is 40${}^{\circ }$. The flapping amplitudes of the three body joints are 45${}^{\circ }$, 50${}^{\circ }$, and 30${}^{\circ }$, respectively. The phase difference between two adjacent dorsoventral joints is 45${}^{\circ }$.

### 4.2. The Test in Forward Dolphin-like Swimming

**Figure 6:**
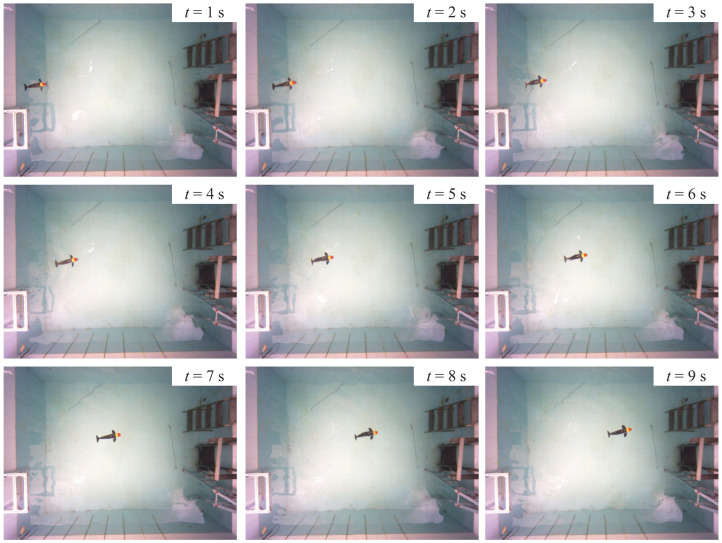
Snapshot of the forward swimming under 1.6 Hz.

**Figure 7:**
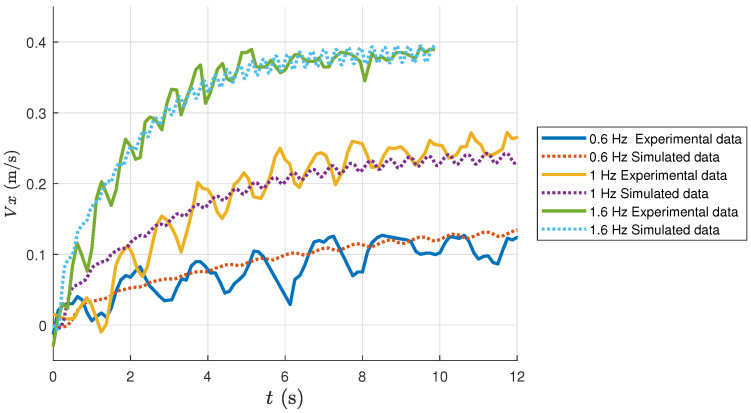
Comparison between the forward swimming velocities in simulation and experiment.

The first experiment is to test the forward swimming capability of the developed robotic dolphin. Specifically, it oscillates its three body joints as well as the flukes to perform dolphin-like swimming, as shown in Figure 6. Figure 7 illustrates the comparison between the simulation results and the experimental results. Intuitively, the dynamic model matches the experimental results well. As the oscillation frequency increases, the swimming velocity of the bionic gliding robotic dolphin increases correspondingly, which is consistent with that of real dolphins [24,25,26]. The maximum swimming velocity of the bionic gliding robotic dolphin was achieved when the oscillation frequency was 1.6 Hz, and the swimming velocity at this time was about 0.38 m/s (0.63 BL/s), basically in line with the simulation results, as shown in Figure 7.

### 4.3. The Test in Turning with the Yaw Joint

**Figure 8:**
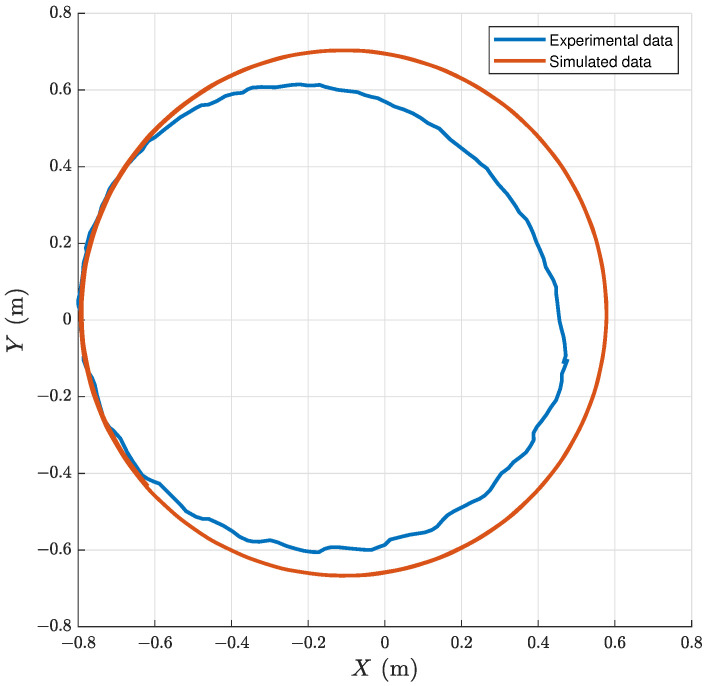
Turning trajectories under an oscillation frequency of 1 Hz.

The second experiment focuses on the turning motion under the help of the yaw joint. From the experimental results, the turning radius of the bionic gliding robotic dolphin is approximately 1.27 m (2.11 BL), 0.65 m (1.08 BL), and 0.53 m (0.88 BL) at frequencies of 0.6 Hz, 1 Hz, and 1.6 Hz, respectively. Meanwhile, the corresponding radii from the simulation results are separately 0.71 m (1.18 BL), 0.69 m (1.14 BL), and 0.67 m (1.12 BL). There is a certain gap between the simulation results and the experimental ones. Figure 8 illustrates the comparison results of the turning radius of the experiments and simulations when the robotic dolphin turns with the yaw joint under an oscillation frequency of 1 Hz. We can see that the real-world turning radius is smaller than simulation one. There are many reasons for this phenomenon here. We know that it is very hard to build an entirely accurate dynamic model for bionic robotic dolphins because of the complex hydrodynamic forces, especially for turning motion. Many unknown factors and perturbations in the real world are omitted or simplified in the simulation model. This leads to a certain gap between the simulation results and the experimental ones.

### 4.4. The Test in Gliding Motion

**Figure 9:**
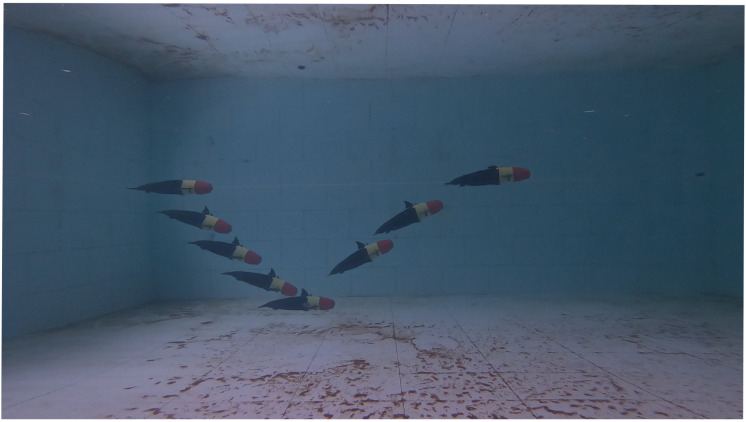
Snapshot of the gliding motion.

**Figure 10:**
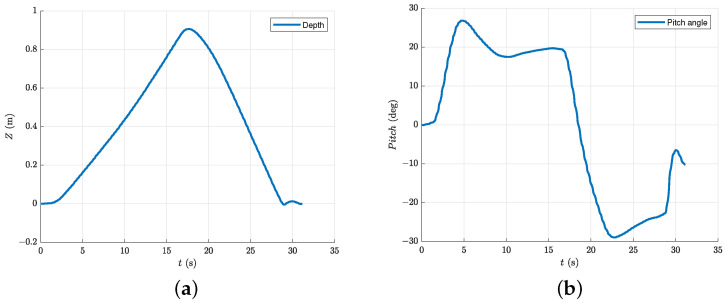
Experimental results of the gliding motion. (**a**) Gliding depth; and (**b**) Pitch angle.

In the following, gliding motion experiments are performed, with video sequences shown in Figure 9 and motion data curves shown in Figure 10. At the beginning of the gliding motion, the buoyancy-driven system moved the piston to the rear for a water-absorbing operation, which resulted in a reduction in the body’s buoyancy. The bionic gliding robotic dolphin gained vertical acceleration and began to glide downward. At the same time, the center of gravity moved forward due to the inhaled water, generating a head-bend torque so that the bionic gliding robotic dolphin acquired a suitable pitch angle. When starting to glide upwards, the buoyancy-driven system needed to drain some water to obtain some upward buoyant force. Meanwhile, the slider moved backwards to generate a head lift moment. Due to the limited experimental space, a single dive-float switching process was selected in this experiment. It was set to perform floating operations at a diving depth of approximately 0.9 m. However, Figure 10a shows that the actual switching depth of the gliding motion is different from the set depth, and the reason is that the velocity does not decrease to 0 during the gliding motion. As the bionic gliding robotic dolphin dived down to 0.85 m, the bionic gliding robotic dolphin began to rise. According to Figure 10a, we can see that the velocity of upward gliding motion is higher than that of the downward gliding motion. One of the reasons is that the thrusts in these two gliding phases are different. In the initial state, the robotic dolphin is not fully immersed, but leaves the dorsal fin protruding through the surface of the water. Therefore, it leads to a different thrust in different gliding phases, i.e., the thrust in the upward gliding motion is greater than that in the downward gliding motion, since the thrusts from the buoyancy regulation mechanism in two gliding phases are equal, i.e., one half of the water injector for gliding downwards and one half for gliding upwards.

Figure 10b illustrates the variation of the pitch angle in the gliding motion. The results show that the steady-state pitch angle is approximately symmetrical in the process of upward and downward gliding motions, and the difference is about 5${}^{\circ }$. There are many reasons to lead to this difference pitch angle, e.g., the different thrust from the dorsal fin not immersed in water, the different drag from the up-down asymmetric body shape and the unsteady state of the upward gliding motion from the limited experimental space.

**Figure 11:**
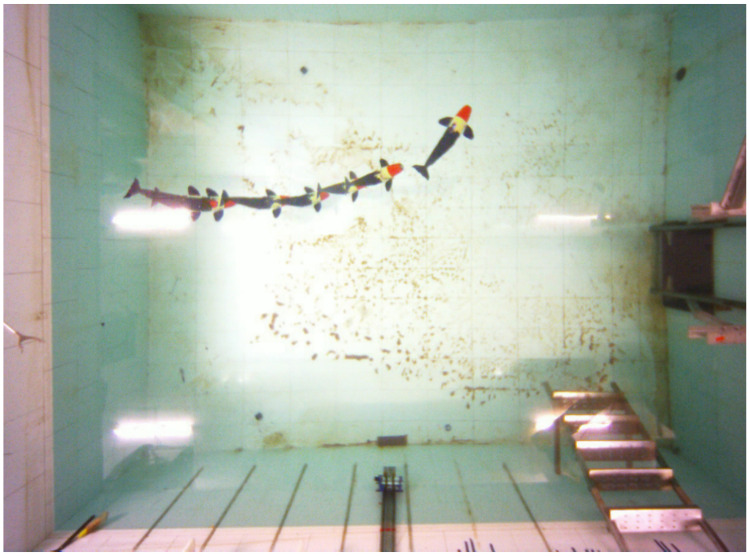
Snapshot of the gliding turning motion with the yaw joint.

**Figure 12:**
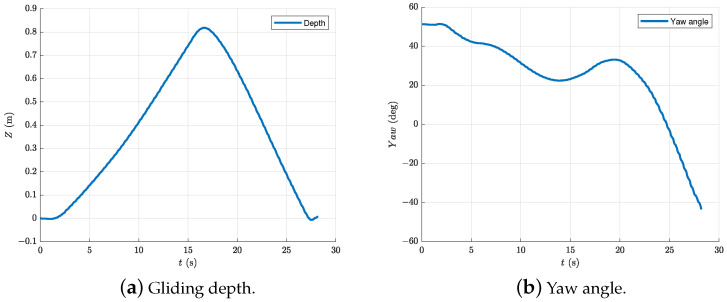
Experiment results of the gliding turning motion with the yaw joint. (**a**) Gliding depth; and (**b**) Pitch angle.

The last experiment focuses on the turning motion of the robotic dolphin in the gliding process. In order to realize turning, the robotic dolphin rotated its yaw joint to 40${}^{\circ }$ during both downward and upward gliding motions, as shown in Figure 11. Figure 12 shows the experimental results of the turning motion in the gliding process. According to Figure 12a, we can see that the robotic dolphin turns in the gliding motion, when the yaw joint is employed, i.e., rotates 30${}^{\circ }$ in this test. More careful observation further reveals that the turning in the upward gliding motion is more obvious than that in the downward gliding motion. For example, in the downward gliding process, the robotic dolphin turns about 60${}^{\circ }$, i.e., from 25${}^{\circ }$ to −45${}^{\circ }$; comparatively, it turns about 25${}^{\circ }$ in the upward gliding process, i.e., from 50${}^{\circ }$ to 25${}^{\circ }$, as shown in Figure 12b. One of the reasons leading to this phenomenon is the different gliding velocities in these two gliding phases. According to Figure 12a, we can see that the robotic dolphin moves faster in the downward gliding motion, compared to the upward gliding motion. Higher gliding velocity produces higher turning moment. Therefore, a turning appears in the downward gliding motions.

### 4.5. Discussion

After analyzing the experimental results, we draw conclusions as follows. For the novel bionic gliding robotic dolphin, the newly added yaw joint had a noticeable effect. In the dolphin-like mode, in the case of turning with the yaw joint, the turning radius decreases as the oscillation frequency increases. At the same time, the swimming velocity is slightly reduced compared to that in forward swimming, even if under the same oscillation frequency. Obviously, during turning motion, an increase in the frontal area of the robotic dolphin enlarges the hydrodynamic drag, and further leads to the decrease of swimming velocity. In the gliding mode, the yaw motion is enabled by the adoption of the yaw joint. Again, the yaw joint has little effect on the velocity of gliding motion.

Regarding to the propulsive performance, there is an obvious gap between the developed robotic dolphin and the real dolphins in nature. Specifically, the developed gliding robotic dolphin finally obtains the maximal swimming velocity of 0.63 BL/s, maximal turning rate of 30${}^{\circ }$/s and minimal turning radius of 0.88 BL. By contrast, the bottlenose dolphin easily achieves the maximal swimming velocity at 7.84 BL/s and the killer whale can realize a turning motion with a turning radius of 0.11 BL and a turning rate of 232.5${}^{\circ }$/s [5]. The main reasons are that the employed servo motors cannot provide such large driven torques and turning speeds as the biological muscles’; meanwhile, the mechanical constraints also lead to much inflexibility. However, as a kind of underwater glider, the developed gliding robotic dolphin obtains a little larger speed than conventional underwater gliders, e.g., Slocum’s 0.13 BL/s and Spray’s 0.13 BL/s [5]. Additionally, compared to the conventional AUVs, the developed robotic dolphin achieves an inferior propulsive speed, but superior turning rate and radius, where most AUVs’ maximal turning rates are less than 10${}^{\circ }$/s, and the minimal radius is more than 2 BL [5].

## 5. Conclusions and Future Work

In this paper, we have developed a novel type of bionic gliding robotic dolphin to realize both dolphin-like swimming for increased maneuverability and gliding motion for long endurance underwater. Considering the poor turning ability of the existing gliding robotic dolphins, we particularly introduced a yaw joint for the developed robotic dolphin to realize enhanced turning maneuvers. Thereafter, relying on the Newton–Euler method, a full-state dynamic model of both dolphin-like swimming and gliding motions is established. Finally, the simulations and experiments verify that the special yaw joint effectively improves the turning maneuverability of the bionic gliding robotic dolphin. This survey establishes a solid theoretical foundation for follow-up studies and provides valuable guidance for the further application of the bionic gliding robotic dolphin.

Future work will focus on investigating combinations of turning patterns by drawing on intelligent algorithms such as reinforcement learning to further achieve path planning and following in the ocean.

## Data Availability

The data generated during the current study are available from the corresponding author on reasonable request.
